# Protective Immunity Induced by Incorporating Multiple Antigenic Proteins of *Toxoplasma gondii* Into Influenza Virus-Like Particles

**DOI:** 10.3389/fimmu.2018.03073

**Published:** 2019-01-07

**Authors:** Su-Hwa Lee, Hae-Ji Kang, Dong-Hun Lee, Fu-Shi Quan

**Affiliations:** ^1^Department of Biomedical Science, Graduate School, Kyung Hee University, Seoul, South Korea; ^2^Department of Medical Zoology, Kyung Hee University School of Medicine, Seoul, South Korea; ^3^Biomedical Science Institute, Kyung Hee University School of Medicine, Seoul, South Korea

**Keywords:** virus-like particle, *Toxoplasma gondii*, vaccine, protection, multi-antigen

## Abstract

Virus-like particle (VLP) as a highly efficient vaccine platform has been used to present single or multiple antigenic proteins. In this study, we generated VLPs (multi-antigen VLPs, TG146) in insect cells co-infected with recombinant baculoviruses presenting IMC, ROP18, and MIC8 of *Toxoplasma gondii* together with influenza matrix protein 1 (M1) as a core protein. We also generated three VLPs expressing IMC, ROP18, or MIC8 together with M1 for combination VLPs (TG1/TG4/TG6). A total of four kinds of VLPs generated were characterized by TEM. Higher number of VLPs particles per μm^2^ were observed in multi-antigen VLPs compared to combination VLPs. Mice (BALB/c) were intranasually immunized with multi-antigen VLPs or combination VLPs and challenged with *T. gondii* tachyzoites (GT1) intraperitoneally. Compared to combination VLPs, multi-antigen VLPs showed significantly higher levels of CD4^+^ T cell, and germinal center B cell responses with reduced apoptosis responses, resulting in significant reduction on parasite burden. These results indicate that higher efficacy of VLPs generated by multi-antigen VLPs can induce significant reduction of parasite burden and better survival of mice than that by combination VLPs, providing important insights into vaccine design strategy for VLPs vaccine expressing multiple antigenic proteins.

## Introduction

Virus-like particle (VLP) is one of the most promising approach for vaccine development. VLPs represent safe vaccine platforms since VLPs are composed of viral structural proteins without requiring the presence of the genome. In addition, VLPs are highly immunogenic, even in the absence of adjuvants compared to soluble subunit protein vaccines (1, 2). VLPs can efficiently activate antigen presenting cells (2, 3) and elicit CD4^+^ and CD8^+^ T cell immune responses, inducing strong stimulation of B cell mediated responses by direct cross-linking of BCRs in B cells (4). *Toxoplasma gondii*, an obligate intracellular protozoan parasite, is widespread throughout the world. It causes toxoplasmosis, a disease of major medical and veterinary importance. It causes congenital disease and abortion in humans and domestic animals. An effective vaccine could be an ideal choice to prevent and control toxoplasmosis.

Previous studies have shown that *T. gondii* VLP vaccines can provide protection against *T. gondii* ME49 strain or GT1 strain using mouse models (5, 6). In these studies, *T. gondii* inner membrane complex (IMC) or microneme protein 8 (MIC8) proteins were expressed on the surface of VLPs. These proteins were selected because they are critical for the proliferation and invasion of host cells. These studies indicate that VLP vaccine immunizations can induce serum antibody responses or serum neutralizing activity and T cell responses to show complete protection (6). *T. gondii* rhoptry protein ROP18 is known as a major virulence factor that regulates intracellular proliferation of *T. gondii* in host cells (7–9). ROP18 has not been studied in VLP form. We hypothesized that VLPs containing multiple proteins of *T. gondii* IMC, MIC8, and ROP18 might induce high vaccine efficacy. A *T. gondii* vaccine encoding multistage antigens is known to confer protective immunity to BALB/c mice against parasite infection (10). Recombinant multi-antigenic vaccines composed of surface and secretory antigens of *T. gondii* in murine models can also induce strong protection against *T. gondii* invasion (11). These studies support our novel hypothesis on vaccine efficacy induced by VLPs containing three antigenic proteins of *T. gondii* (IMC, MIC8, ROP18). Multi-protein vaccines might induce immunity against multiple antigenic targets, multiple strain variants, and/or even multiple pathogens. High immunity induced by multiple antigenic proteins expressed by VLPs could be promising alternative screening strategy.

There are two ways to generate VLPs containing multiple antigenic proteins. One is through combination VLPs in which VLPs expressing three antigens can be generated by combining three VLPs with each VLP produced by infecting insect cells with baculoviruses expressing single antigenic protein. Another is through multi-antigen VLPs that can be generated by infecting insect cells simultaneously with three baculoviruses with each baculovirus expressing a single antigenic protein (multi-antigen VLPs). The process in producing multi-antigen VLPs is much easier and faster than that from combination VLPs. Thus, in this study, we evaluated and compared protective immune responses induced by multi-antigen VLPs with combination VLPs expressing IMC, MIC8, and ROP18 proteins. Multi-antigen VLPs showed better protection than combination VLPs by inducing significantly higher levels of T cell and germinal center B cell responses with reduced apoptosis responses, resulting in significant reduction of parasite burden.

## Materials and Methods

### Ethics Statement

All animal experiments and husbandry involved in these studies were conducted under the guidelines of Kyung Hee University IACUC (permit number: KHUASP (SE)−16–157). All animal procedures performed in this study were reviewed, approved, and supervised by Kyung Hee University IACUC. The research staff was trained in animal care and handling. They have received the certificate of completion for Animal Welfare & Ethics Course (K-2015-18060371) form CITI. All surgery was performed under isoflurane anesthesia (BSL2). All efforts were made to minimize animal suffering.

### Animals, Parasites, Cells, and Antibodies

*T. gondii* GT1 strains were maintained in female BALB/c obtained from KOA Tech (Pyeogtaek, South Korea) as described previously (5, 6, 12, 13). *T. gondii* GT1 and ME49 strains were maintained by serial intraperitoneal passage (GT1) or oral passage (ME49) in Balb/C mice as described (5). *Spodoptera frugiperda* Sf9 cells used for production of recombinant baculovirus (rBV) and virus-like particles were maintained in serum-free SF900 II medium (Invitrogen, Carlsbad, CA, USA) in spinner flasks at 27°C and 130–140 rpm. *T. gondii* (ME49)–infected sera from mice were used. Horseradish peroxidase (HRP)-conjugated goat anti-mouse immunoglobulin IgG was purchased from Southern Biotech (Birmingham, AL, USA). Monoclonal mouse anti-M1 antibody was purchased from Abcam (Cambridge, UK).

### Generation of Plasmid, Recombinant Baculovirus, and Virus-Like Particles

For plasmid constructions, *T. gondii* IMC, rhoptry protein 18 (ROP18), microneme protein 8 (MIC8), and influenza matrix protein 1 (M1) genes were clones as described previously (5, 6). These constructs containing IMC (accession number: HQ012579, 495 bp), ROP18 (accession number: AM075204, 1,665 bp), MIC8 (accession number: AF353165, 2,055 bp), or M1 (accession number: EF467824, 1,027 bp) gene in pFastBac vector were confirmed by DNA sequencing. Recombinant plasmids were transformed into DH10-Bac. Recombinant baculoviruses expressing IMC, ROP18, MIC8, or M1 were produced as described previously (5, 6). To produce combination VLPs expressing three proteins (IMC, MIC8, and ROP18), three VLPs were generated with each VLP expressing IMC, MIC8, or ROP18 together with influenza M1 as core protein as described previously (5, 6). To produce multi-antigen VLPs, Sf9 cells were simultaneously co-infected with recombinant baculoviruses together with influenza M1 as core protein. Sf9 cell culture supernatants were collected on day 3 post-infection and cleared by centrifugation at 6,000 rpm for 30 min at 4°C to remove cells. VLPs were purified as described previously (5, 6). VLPs were stored at 4°C until used.

### Characterization of VLPs

Purified VLPs were negatively stained on the grid and observed under a transmission electron microscope (TEM) (JEOL 2100, JEOL USA, Inc.; Peabody, MA, USA) (14). Three grids were stained for each sample and 20–25 regions of the grid images were captured for analysis. These VLPs were characterized by western blot as described previously (6). Briefly, proteins from SDS-PAGE gel were transferred onto PVDF membrane (Millipore) and probed with primary antibody followed by incubation with secondary antibody (HRP-conjugated anti-mouse IgG). The amount of M1 protein was determined with monoclonal mouse anti-M1 antibody.

### Mice Immunization and Challenge

BALB/c mice (female, 7 weeks old) were randomly divided into different experimental groups (*n* = 20 per group) to receive multi-antigen VLPs or combination VLPs. For multi-antigen VLPs (TG146) immunization, 60 μg of multi-antigen VLPs per mouse were used to intranasally (IN) immunize mice at weeks 0 and 4. For combination VLPs (TG1/TG4/TG6), three VLPs expressing IMC, ROP18, or MIC8 were combined at a ratio of 1:1:1 (20 μg from each VLP, total 60 μg protein). At 4 weeks after the 2nd immunization, mice were challenged with 1 × 10^3^ tachyzoites of the GT1 strain by intraperitoneal (IP) injection. Ten mice from each group were sacrificed at 7 days post-challenge. Ascites of abdominal cavity and spleen samples were collected. The remaining mice (10 mice in each group) were observed daily to monitor changes in body weight and survival rates until death. Mice that lost 20% in body weight were humanly euthanized.

### *T. gondii*-Specific IgG Antibody Response

Mice sera were collected from all groups at 4 weeks after prime and boost immunization. Sera from naïve mice were used as a negative control, and sera from *T. gondii* (ME49)—infected sera were used as a positive control. *T. gondii*-specific IgG antibody was determined by enzyme-linked immunosorbent assay (ELISA) as described previously (6). Briefly, 96-well flat-bottom immunoplate were coated with 100 μL of *T. gondii* antigen at a final concentration of 0.5 μg/mL in 0.05 M carbonate bicarbonate buffer (pH 9.6) per well at 4°C overnight. Then 100 μL of serum samples (diluted 1:100 in PBST) per well were incubated in the plates for 2 h at 37°C as primary antibody response. HRP-conjugated goat anti-mouse IgG in PBST (100 μL/well, diluted 1:2,000 in PBST) were used to determine *T. gondii*-specific IgG response.

### Antibody Neutralizing Activity

Mouse sera were collected at week 4 after boost immunization and complement inactivated at 56°C for 30 min. Then 50 μL of sera from immunization was incubated with 100 tachyzoites of *T. gondii* (GT1) at 37°C for 1 h. The mixture of tachyzoites and serum was used to intraperitoneally (IP) infect naive mice (10 mice in each group). The mixture of tachyzoites and PBS was used as control. At 7 days after infection, tachyzoites of *T. gondii* were collected from the abdominal cavity of mouse and counted by hemocytometer chamber under microscopy (15, 16).

### T Cells and Germinal Center B Cell Responses by Flow Cytometry

The population of T cell (CD4^+^ and CD8^+^) and germinal center B cells (GC) from splenocytes of mice on day 7 after challenge infection were analyzed by flow cytometry. Briefly, 1 × 10^6^ splenocytes (each tube) in staining buffer (2% bovine serum albumin and 0.1% sodium azide in 0.1 M PBS) were incubated at 4°C for 15 min with Fc Block (clone 2.4G2; BD Biosciences, CA, USA). For surface staining, cells were incubated with surface antibodies (CD3e-PE-Cy5, CD4-FITC, CD8a-PE, B220-FITC, GL7-PE; BD Biosciences, CA, USA) at 4°C for 30 min. Splenocytes were washed with staining buffer and fixed with 4% paraformaldehyde at 4°C for 30 min before acquisition using a BD Accuri C6 Flow Cytometer (BD Biosciences, CA, USA). Data were analyzed using C6 Analysis software (BD Biosciences, CA, USA).

### Apoptosis Analysis

To determine apoptosis in splenocytes, Annexin-V and PI were stained using BD Apoptosis Detection Kit I (BD Biosciences, CA, USA). Splenocytes were collected at 7 days post-challenge. Then 1 × 10^5^ cell in binding buffer were centrifuged at 400 × g for 10 min and the supernatant was discarded. Cells were stained with 5 μl Annexin V-FITC and PI at room temperature for 15 min in the dark. The number of apoptotic cell was determined using a BD Accuri C6 Folw Cytometer (BD Biosciences, CA, USA) and analyzed with C6 Analysis Software (BD Biosciences, CA, USA).

### Statistics

The data were analyzed statistically using the ANOVA with multiple comparison test of PC-SAS 9.3 (SAS Institute; Cary, NC, USA). A *P* < 0.01 was considered to be significant.

## Results

### Generation of Virus-Like Particles

**Figure 1:**
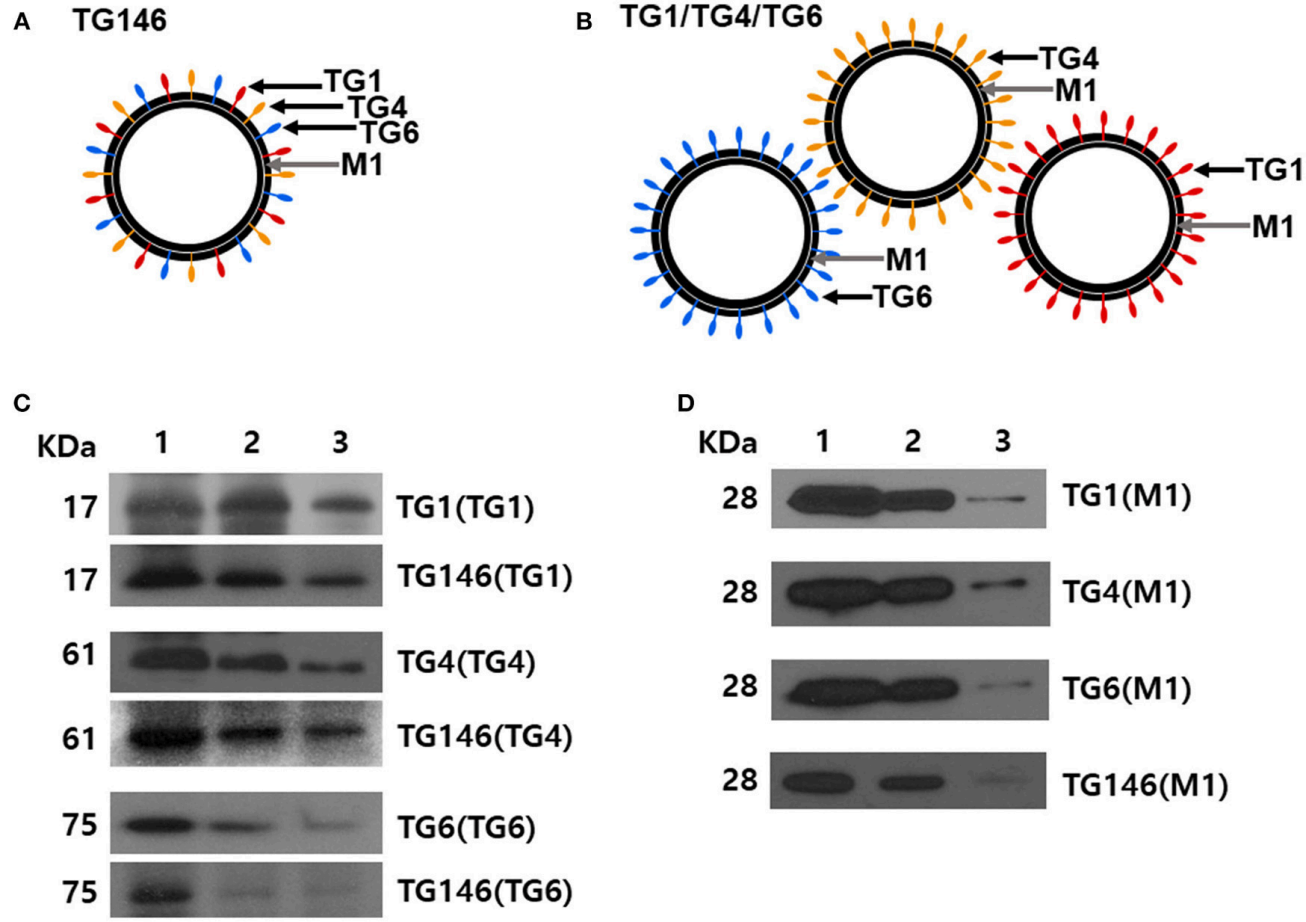
Diagrams for multi-antigen VLPs (TG146 VLPs, **A**) and combination VLPs (TG1/TG4/TG6 VLPs, **B**). Multi-antigen VLPs is a vaccine formulation in which VLPs are generated by simultaneously infecting Sf9 cells with three baculoviruses expressing TG1, TG4, and TG6 proteins together with influenza M1. Combination VLPs is a vaccine formulation in which the exact same amounts of VLPs from TG1 VLPs, TG4 VLPs, and TG6 VLPs are combined. Western blot analyses for IMC (TG1), ROP18 (TG4), and MIC8 (TG6) from *T. gondii* and influenza M1 were performed to determine incorporation into VLPs **(C,D)**. Multi-antigen VLPs **(C)** and combination VLPs **(D)** were loaded for SDS-PAGE. Polyclonal mouse anti-*T. gondii* antibody and anti-M1 monoclonal antibody were used to probe *T. gondii* IMC (TG1), ROP18 (TG4), MIC8 (TG6) protein, and influenza M1 protein. VLPs protein concentrations for Lane 1: 27 μg, lane 2: 9 μg and, lane 3: 3 μg were used.

**Figure 2:**
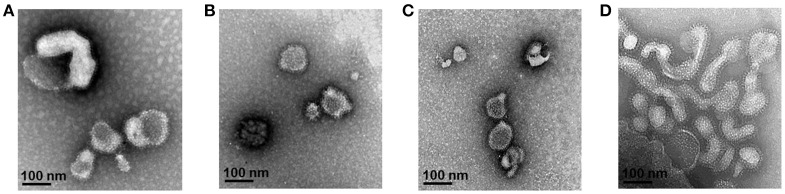
Electron microscopy of *T. gondii* IMC VLPs **(A)**, ROP18 VLPs **(B)**, MIC8 VLPs **(C)**, and multi-antigen VLPs **(D)**. Spikes representing *T. gondii* IMC, ROP18, and MIC8 proteins from single or multi-antigen proteins were observed on the surface of VLPs.

Multi-antigen VLPs or combination VLPs expressing *T. gondii* IMC, ROP18, or MIC8 together with influenza M1 were generated (Figure 1). Figures 1A,B are diagrams showing components of multi-antigen VLPs (A) and combination VLPs (B). IMC, ROP18, and MIC8 protein expression were confirmed by western blot (Figures 1C,D) and electron microscopy (Figure 2). Results indicated that *T. gondii* IMC, ROP18, MIC8, and influenza M1 were incorporated into multi-antigen VLPs (C) or combination VLPs (D). *T. gondii* VLPs had a spherical morphology with a size of 40–120 nm (Figure 2). They exhibited antigen spikes on their surfaces as incorporated protein (Figure 2) as seen in the previous studies (6, 17, 18). In addition, virus-like particles per μm^2^ were counted under microscopy. Importantly, multi-antigen VLPs showed higher number of VLP particles per unit area compared to combination VLPs (Table 1, ^**^P < 0.0004).

**Table 1:** Virus-like particles (VLPs) counts under electron microscopy.

**Virus-like particles**	**Particles (per μm^**2**^)**	**Statistics (*P* < 0.0004)**
TG1 VLPs	15.2 ± 8.4	TG1 vs. Multi^**^
TG4 VLPs	13.3 ± 4.7	TG4 vs. Multi^**^
TG6 VLPs	8.6 ± 1.9	TG6 vs. Multi^**^
Combination VLPs (TG1/TG4/TG6 VLPs)	12.4 ± 6.2	Comb vs. Multi^**^
Multi-antigen VLPs (TG146 VLPs)	22.9 ± 9.4	

Virus-like particles were counted under transmission electron microscopy (TEM). Combination VLPs (TG1/TG4/TG6) expressing three proteins (TG1 for IMC, TG4 for ROP, and TG6) were generated with each VLP expressing IMC, MIC8, or ROP18 together with influenza M1 as core protein as described in materials section. Multi-antigen VLPs (TG146 VLPs) were generated by co-infecting Sf9 cells with rBVs expressing TG1, TG4, and TG6 proteins together with influenza M1 as core protein as described in materials section. Significantly higher number of particles was found in multi-antigen VLPs compared to combination VLPs or single VLPs (TG146 VLPs vs. TG1/TG4/TG6 VLPs, TG1 VLPs, TG4 VLPs, TG6 VLPs,

** *P < 0.0004)*.

### *T. gondii*-Specific IgG Antibody Response

**Figure 3:**
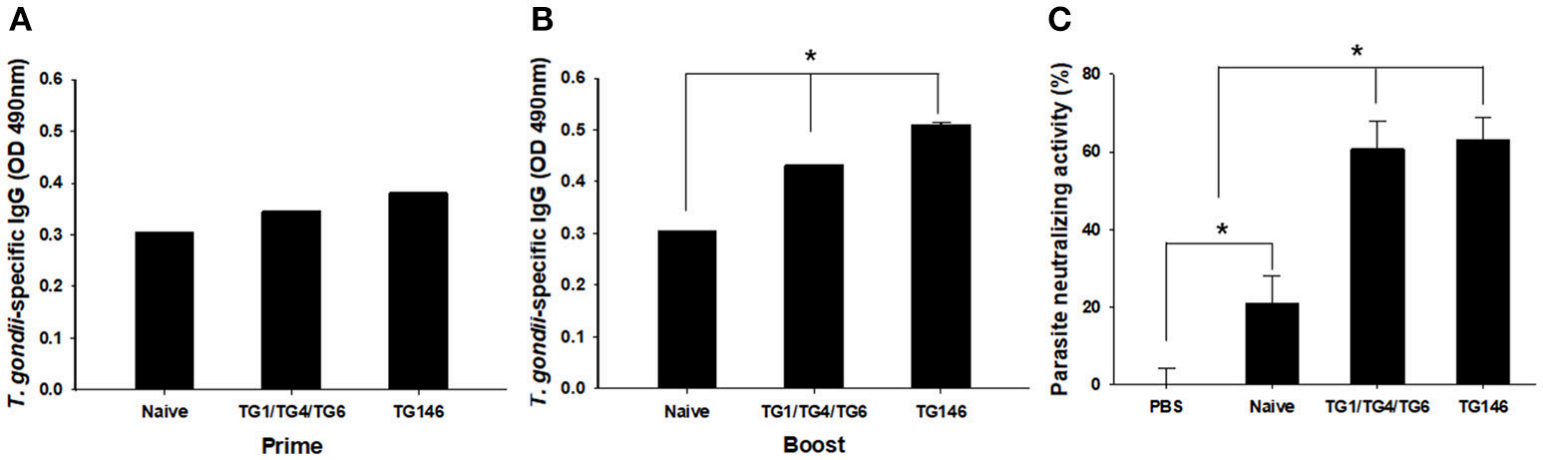
*T. gondii*—specific IgG antibodies and neutralizing activity. *T. gondii*—specific IgG antibodies were induced after prime **(A)** and boost immunization (**B**, ^*^P < 0.05). Sera neutralizing activity was determined using sera from VLPs immunized mice (**C**, ^*^P < 0.01). Both sera from combination VLPs (TG1/TG4/TG6) and multi-antigen VLPs (TG146) significantly inhibited *T. gondii* (GT1) replication compared to PBS control and sera from naïve mice.

To evaluate the level of *T. gondii*-specific IgG antibody induced by multi-antigen VLPs or combination VLPs immunizations, we measured these levels in mice sera upon prime and boost after IN immunizations (Figures 3A,B). Multi-antigen VLPs (TG146) group showed higher levels of *T. gondii*-specific IgG antibody response after the 2nd immunization compared to combination VLPs (TG1/TG4/TG6) group (Figure 3B, ^*^P < 0.01).

### Parasite Neutralizing Activity

Neutralizing activity afford by prior vaccination has long been considered the main mechanism of protection (6). In this study, mice immune sera collected were used to determine parasite neutralizing activity. As shown in Figure 3C, at 7 days after infection, tachyzoites from abdominal cavities of mice were calculated. Counts of tachyzoites in sera from combination VLPs (^*^P < 0.01) or multi-antigen VLPs were significantly reduced (^*^P < 0.01) compared to those in controls (non-immunized naïve sera and PBS). There was no significant difference in parasite neutralizing ability between sera from multi-antigen VLPs and combination VLPs groups.

### Immune Cell Responses

**Figure 4:**
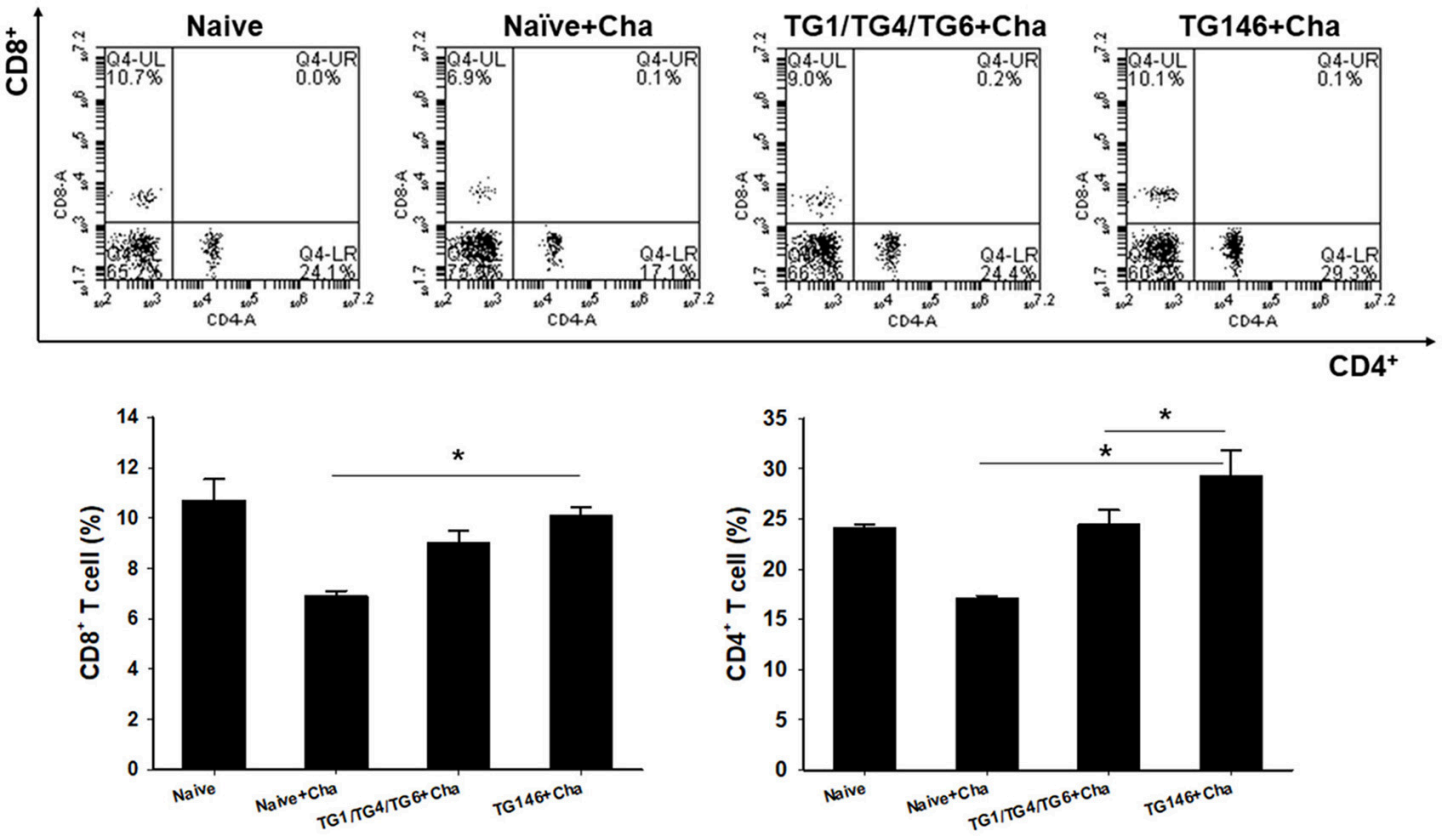
CD4^+^ and CD8^+^ T cell responses in spleen. Populations of CD4^+^ and CD8^+^ T cells were analyzed at 7 days after challenge using flow cytometry. Numbers indicate percentages of cell populations in each quadrant. Both multi-antigen VLPs and combination VLPs showed higher CD4^+^ and CD8^+^ T cell responses compared to non-immunized naïve control. Higher populations of CD4^+^ T cell were detected in multi-antigen VLPs immunized mice compared to those in combination VLPs immunized mice (^*^P < 0.01). Naïve+Cha: Naïve mice were challenge infected with *T. gondii* tachyzoites. TG1/TG4/TG6+Cha: Combination VLPs immunized mice were challenge infected with *T. gondii* tachyzoites. TG146+Cha: multi-antigen VLPs immunized mice were challenge infected with *T. gondii* tachyzoites.

**Figure 5:**
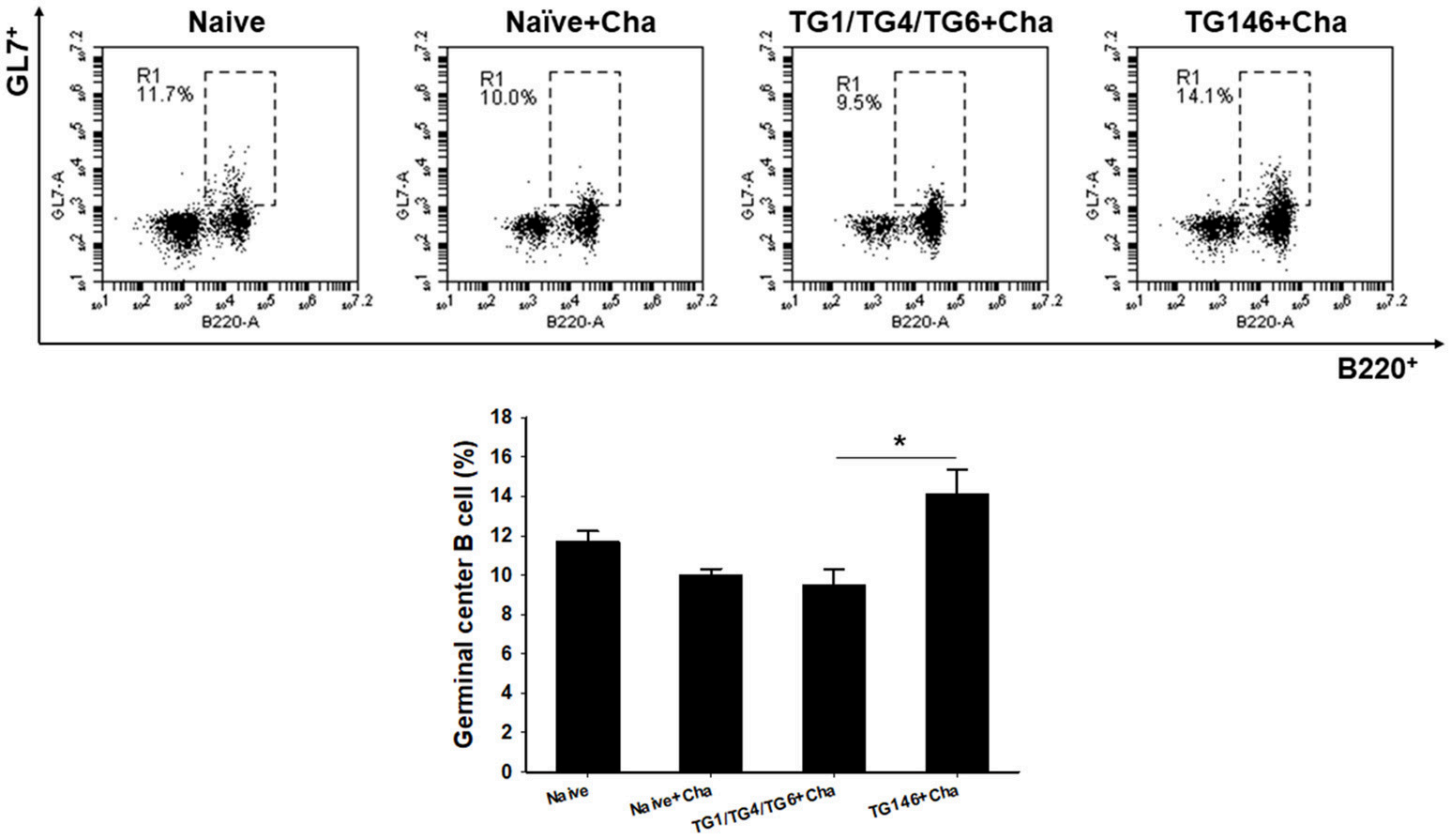
Germinal center B cell response. Populations of germinal center B cell were analyzed at 7 days after challenge using flow cytometry. Higher populations of germinal center B cell were detected in multi-antigen VLPs immunized mice compared to those in combination VLPs (^*^P < 0.01). Naïve+Cha: Naïve mice were challenge infected with *T. gondii* tachyzoites. TG1/TG4/TG6+Cha: Combination VLPs immunized mice were challenge infected with *T. gondii* tachyzoites. TG146+Cha: multi-antigen VLPs immunized mice were challenge infected with *T. gondii* tachyzoites.

To determine T cell and B cell responses in immunized mice upon challenge, flow cytometer analysis was performed using mouse spleen cells. As shown in Figures 4, 5, at 7 days post-challenge, higher populations of CD4^+^ T cells and CD8^+^ T cells (Figure 4) were found in multi-antigen VLPs (TG146+Cha) (^*^P < 0.01) or combination VLPs (TG1/TG4/TG6+Cha) (^*^P < 0.01) immunized mice compared to naïve challenge (Naïve+Cha) control mice. Significantly higher levels of CD4^+^ T cell responses were also found in multi-antigen VLPs compared to those in combination VLPs (^*^P < 0.01). However, there was no significant difference in CD8^+^ T cell responses between multi-antigen VLPs and combination VLPs. As shown in Figure 5, multi-antigen VLPs showed significant higher levels of germinal center B cells responses compared to combination VLPs (^*^P < 0.01).

### Apoptotic Response

**Figure 6:**
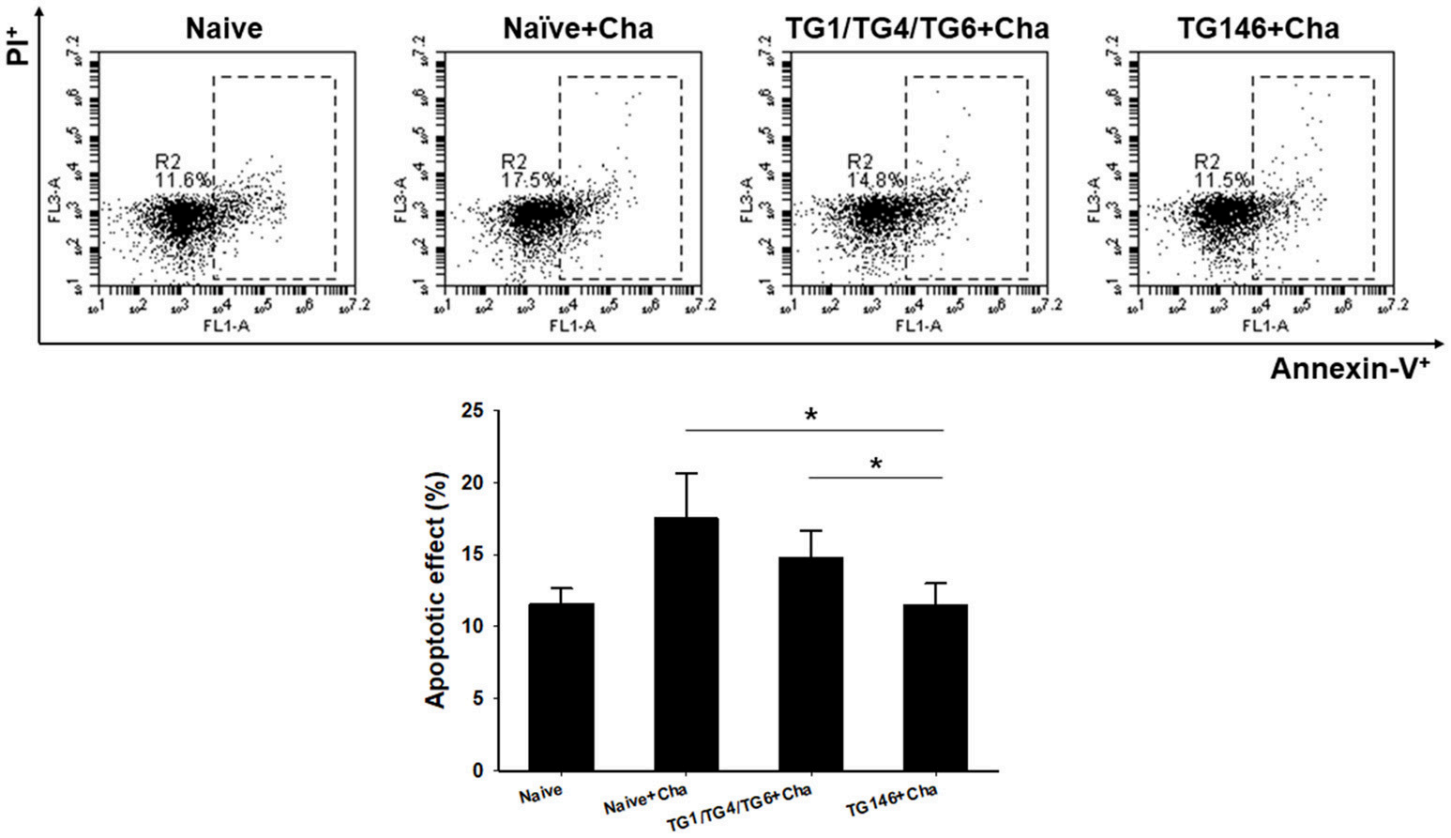
Apoptotic responses upon challenge infection. To measure apoptotic response in the spleen, mice splenocytes were stained with AnnexinV-FITC and PI. Significantly higher levels of apoptotic responses were found in Naïve+Cha and combination VLPs immunized mice compared to those in multi-antigen VLPs immunized mice (^*^P < 0.01). Naïve+Cha: Naïve mice were challenge infected with *T. gondii* tachyzoites. TG1/TG4/TG6+Cha: Combination VLPs immunized mice were challenge infected with *T. gondii* tachyzoites. TG146+Cha: multi-antigen VLPs immunized mice were challenge infected with *T. gondii* tachyzoites.

*T. gondii* is highly pathogenic. It induces high level of apoptosis, leading to widespread parasite dissemination and rapid death of mice (19). Mouse splenocytes were collected and apoptosis response was analyzed by FACS. As shown in Figure 6, significantly higher levels of apoptotic responses were found in non-immunized naïve challenge control (^*^P < 0.01) while multi-antigen VLPs (^*^P < 0.01) or combination VLPs (^*^P < 0.01) resulted in significantly lower levels of apoptotic responses. Compared to combination VLPs, multi-antigen VLPs showed significantly lower level of apoptotic responses (^*^P < 0.01).

### Survival Rate, Body Weight Changes, and Parasite Burden Against Challenge Infection With *T. gondii*

**Figure 7:**
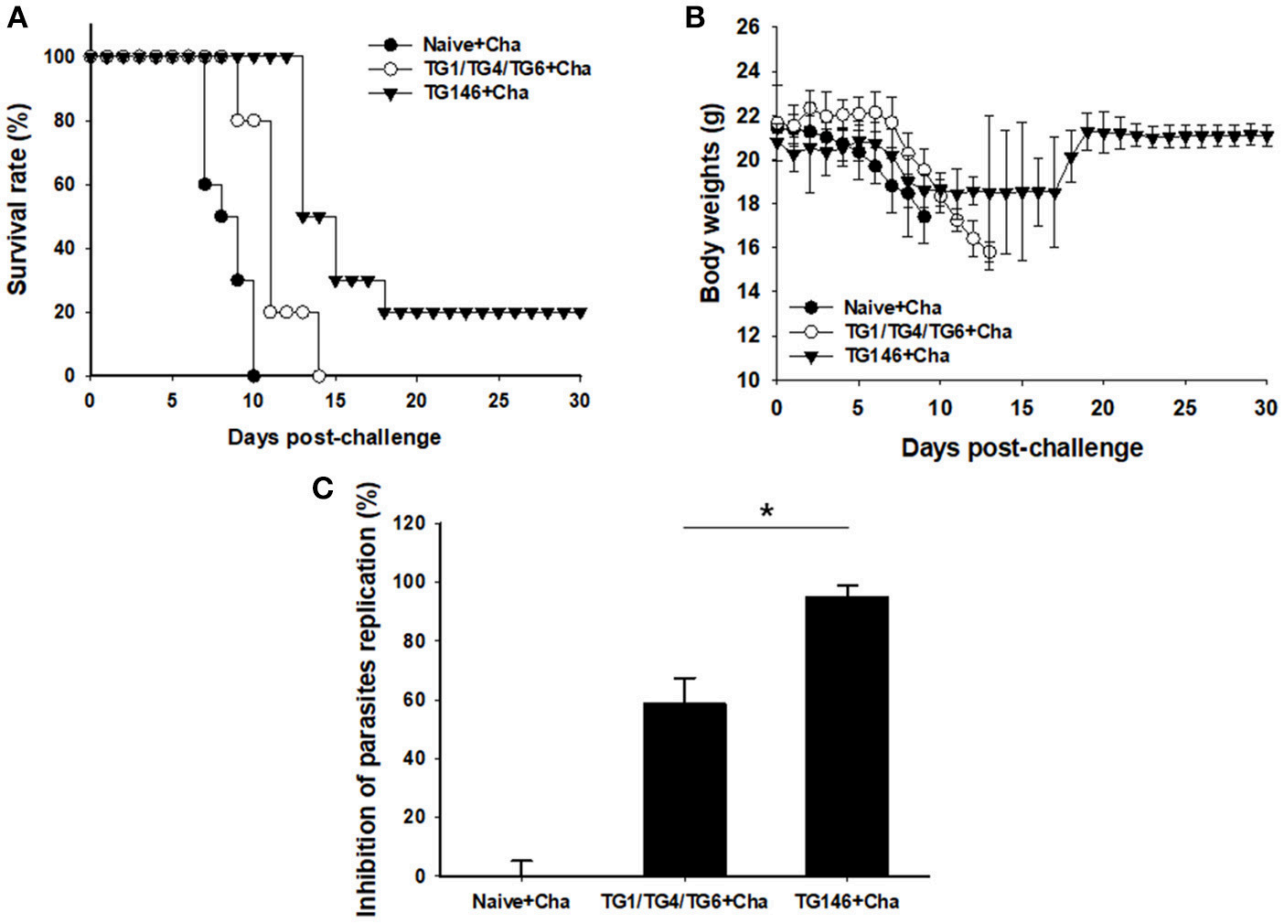
Survival rate, body weight changes, and parasite burden upon challenge infection. Mice immunized with combination VLPs (TG1/TG4/TG6), multi-antigen VLPs (TG146), and non-immunized control mice were challenged with 1 × 10^3^ tachyzoites of *T. gondii* GT1 strain. Survival rate and body weight were monitored daily for 30 days after challenge infections. All control mice (Naïve+Cha) died within 10 days whereas mice immunized with multi-antigen VLPs (TG146 Cha) showed a survival rate of 20% **(A)**. Also, surviving mice (TG146 Cha) retained their recovered body weights from day 19 after challenge infection **(B)**. Parasite burden **(C)** was counted to determine parasite replication. Mice immunized with multi-antigen VLPs or combination VLPs showed significant reductions in *T. gondii* replication compared to non-immunized control mice at 7 days after challenge infection (^*^P < 0.01). Naïve+Cha: Naïve mice were challenge infected with *T. gondii* tachyzoites. TG1/TG4/TG6+Cha: Combination VLPs immunized mice were challenge infected with *T. gondii* tachyzoites. TG146+Cha: multi-antigen VLPs immunized mice were challenge infected with *T. gondii* tachyzoites.

To determine the protective efficacy of VLPs vaccine, immunized, and control mice were challenged with lethal *T. gondii* GT1 strain (1 × 10^3^ tachyzoites) at 4 weeks after boost by IP injection. Survival rates and protection were determined. As shown in Figure 7A, multi-antigen VLPs vaccine (TG146+Cha) significantly increased the survival rate (20%) compared to other groups (Naïve+Cha, TG1/TG4/TG6+Cha) in which all mice died within 14 days after challenge. Mice that were immunized with multi-antigen VLPs showed 10.5% body weight loss at day 11 whereas mice that received combination VLPs and naïve mice showed 21.5% at day 13 and 16.8% at day 9, respectively (Figure 7B). To assess VLP vaccine efficacy, at 7 days after challenge infection, tachyzoites of *T. gondii* were collected from abdominal cavities of mice and counted. Mice immunized with multi-antigen VLPs or combination VLPs significantly inhibited parasite replication compared to non-immunized control (Naïve+Cha) (Figure 7C, ^*^P < 0.01). Multi-antigen VLPs showed higher inhibition of parasite replication compared to combination VLPs (^*^P < 0.01).

## Discussion

*T. gondii* has a complex and variety of invasion pathways for attaching and invading host cells. Although several invasion factors of *T. gondii* involved in invasion of host cells have been identified as vaccine candidate antigens, those capable of inducing a strong protective immunity are limited (20). Thus, it is essential to develop multi-antigenic vaccines to overcome the deficiency of using a single antigen as vaccine candidate. *T. gondii* IMC is involved in critical processes such as host cell invasion and daughter cell formation (21). MIC8 proteins play an important role in the invasion process of apicomplexan parasites through adhesion to host cells (22, 23). ROP18 is involved in *T. gondii* invasion and host cell interaction (24). We hypothesized that IMC, MIC8, or ROP18 antigens in VLPs platforms can induce high levels of vaccine efficacy. Effective vaccines targeting multiple invasion factors may contribute to the prevention and control of toxoplasmosis. Thus, in this study, we targeted *T. gondii* multiple antigens IMC, ROP18, and MIC8 and generated VLPs using SF9 cells with recombinant baculoviruses expressing IMC, ROP18, or MIC8. VLP vaccines targeting multiple antigens by baculovirus expression system could be produced by different ways. In this study, we generated two kinds of VLPs: multi-antigen VLPs and combination VLPs. Multi-antigen VLPs were produced by simultaneously infecting Sf9 cells with three different baculoviruses expressing IMC, ROP18, or MIC8. This way was much more rapid and convenient since only one bottle was needed to prepare VLPs expressing three proteins. Preparation of combination VLPs was complicated since it involved combination of three single VLPs expressing IMC, ROP18, or MIC8 protein independently. In this case, to generated VLPs, three bottles were used, thus needing 3-fold time, and materials during the process of VLPs ultracentrifugation, purification, and dialysis. Importantly, we found that multi-antigen VLPs showed higher density or purity of particles per μm^2^ observed by electronic microscopy than combination VLPs. The strategy of multi-antigen VLPs preparation would have significant impact on designing and preparing VLPs expressing multiple antigens.

Neutralizing antibodies play an important role in protection against viral infection *in vivo* (25–27). In these studies, viral neutralizing activity was determined by infectivity using *in vitro* cell cultures. However, in our current study, we determined the infectivity of *T. gondii* in mice (*in vivo*) induced by serum neutralizing activity to obtain more convincing results. Both multi-antigen VLPs and combination VLPs showed higher levels of neutralizing activity compared to naïve control. No significant difference in parasite neutralizing ability between multi-antigen VLPs and combination VLPs were determined. However, interestingly, higher levels of *T. gondii*-specific IgG antibody responses in sera from multi-antigen VLPs vaccination than those in sera from combination VLPs were found, indicating non-neutralizing antibodies from multi-antigen VLPs might be involved in reduction of parasite burden. In the current study, significantly higher levels of CD4^+^, CD8^+^ T cell, and B cell responses were observed. Importantly, multi-antigen VLPs showed higher levels of responses compared to combination VLPs, indicating that higher density or higher purity of multi-antigen VLPs might contribute to higher immunogenicity and better protection compared to combination VLPs.

*T. gondii* GT1 is highly pathogenic and uniformly lethal. GT1 infections by IP route can lead to complete destruction and cell death in the spleen, resulting in remarkable high levels of IFN-g production and death of mice (19). In this study, both multi-antigen VLPs and combination VLPs showed lower apoptotic responses compared to naïve challenge control. In particularly, multi-antigen VLPs showed similar level of apoptotic response to naïve control. This may be associated with its better protection than combination VLPs by showing increased survival and parasite reduction.

## Conclusion

In summary, multi-antigen VLPs showed high quality of particles and significantly saved time for VLPs preparation compared to combination VLPs. Higher quality of multi-antigen VLPs induced higher T and B cell responses and significantly reduced apoptotic responses, resulting in significant higher inhibition of parasite replication and increased survival rate compared to combination VLPs. These results provide very important insights into vaccine design strategy for *T. gondii* VLPs expressing multiple antigenic proteins.

## Author Contributions

S-HL carried out the molecular genetic studies, participated in the sequence alignment, prepared VLPs, animal experiment and drafted the manuscript. H-JK and D-HL carried out experiment. F-SQ carried out the experiment design, analysis and interpretation of data and drafted the manuscript. All authors read and approved the final version of the manuscript.

### Conflict of Interest Statement

The authors declare that the research was conducted in the absence of any commercial or financial relationships that could be construed as a potential conflict of interest.

## Abbreviations

TG1: *Toxoplasma gondii* inner membrane complex (IMC)
TG4: *Toxoplasma gondii* rhoptry protein 18 (ROP18)
TG6: *Toxoplasma gondii* microneme protein 8 (MIC8)
TG146 VLPs: multi-antigen VLPs
TG1/TG4/TG6 VLPs: combination VLPs.
